# Supplementary material for: Stitchr: stitching coding TCR nucleotide sequences from V/J/CDR3 information
Source: Nucleic Acids Res. 2022 Mar 24;50(12):e68. doi: 10.1093/nar/gkac190 (PMC9262623; doi:10.1093/nar/gkac190)
Supplement: gkac190_Supplemental_File [file gkac190_supplemental_file.pdf]

Supplementary Tables

TCR clone	HLA allele	Peptide	Source	HLA-matched target line	Publication DOI	PDB accession
**MAG-IC3**	A*01:01	EVDPIGHLY	MAGE-A3 (Human)	MDA-MB-436	10.1038/srep18851	5BRZ
**C1-28**	A*24:02	RFPLTFGWCF	Nef (HIV)	SW620	10.1038/srep03097	3VXM
**KFJ5**	B*07:02	APRGPHGGAASGL	NY-ESO-1 (Human)	Karpas-299	10.1038/s41467-018-03321-w	6AVF
**LC13**	B*08:01	FLRGRAYGL	EBNA2A (EBV)	MDA-MB-436	10.1016/S1074-7613(02)00513-7	1MI5
**D2H**	A*11:01	GTSGSPIVNR	NS3 (DENV)	Karpas-299	10.1038/ni.3850	-

Supplementary Table 1: Antigen and reference details for the TCRs used for low-throughput Stitchr validation in Figure 2. Cell lines that do not express the relevant HLA class I allele were used as the HLA negative controls for other TCRs, as well as additional cell lines (e.g. SK-N-SH and SU-DHL-1, which were used as negative control targets for the KFJ5 TCR).

TCR clone	TRAV	TRAJ	TRA CDR3	TRBV	TRBJ	TRB CDR3
**MAG-IC3**	TRAV21*02	TRAJ28	CAVRPGGAGPFFVVF	TRBV5-1	TRBJ2-7	CASSFNMATGQYF
**C1-28**	TRAV8-3	TRAJ28	CAVGAPSGAGSYQLTF	TRBV4-1	TRBJ2-7	CASSPTSGIYEQYF
**KFJ5**	TRAV4	TRAJ21	CLVGEILDNFNKFYF	TRBV28	TRBJ2-3	CASSQRQEGDTQYF
**LC13**	TRAV26-2	TRAJ52	CILPLAGGTSYGKLTF	TRBV7-8	TRBJ2-7	CASSLGQAYEQYF
**D2H**	TRAV9-2	TRAJ37	CALDSGNTGKLIF	TRBV11-2	TRBJ2-7	CASTTGGGGYEQYF

Supplementary Table 2: TCR rearrangement details for TCRs used for low-throughput Stitchr validation in Figure 2. Note that unless specified, all TCR alleles were *01 for their respective genes.

Common Name	Genus species	TRA	TRB	TRG	TRD
**CAT**	*Felis catus*	✔	✔	✔	✔
**COW**	*Bos taurus*	✔	✔	✔	✔
**CYNOMOLGUS_MONKEY**	*Macaca fascicularis*		✔		
**DOG**	*Canis lupus familiaris*	✔	✔	✔	✔
**DOLPHIN**	*Tursiops truncatus*	✔		✔	✔
**DROMEDARY**	*Camelus dromedarius*		✔	✔	
**FERRET**	*Mustela putorius furo*		✔		
**HUMAN**	*Homo sapiens*	✔	✔	✔	✔
**MOUSE**	*Mus musculus*	✔	✔	✔	✔
**NAKED_MOLE-RAT**	*Heterocephalus glaber*	✔	✔	✔	✔
**PIG**	*Sus scrofa*		✔		
**RABBIT**	*Oryctolagus cuniculus*		✔	✔	✔
**RHESUS_MONKEY**	*Macaca mulatta*	✔	✔	✔	✔
**SHEEP**	*Ovis aries*	✔	✔		✔

Supplementary Table 3: Species and loci which are currently ‘stitchable’. These are the loci for which IMGT/GENE-DB has sufficient data available (i.e. at least one leader, variable, joining, and constant region sequence for a given locus).

Gene	Closest Allele	Inferred Allele	Number of donors	Omer & Peres et al?
TRAV12-1	*01	C147G	2	
TRAV12-1	*01	T165C	1	
TRAV12-2	*02	A166C	2	
TRAV14/DV4	*02	A102C	3	
TRAV14/DV4	*02	G223A	1	
TRAV25	*01	G51A	11	
TRAV25	*01	A79G	1	
TRAV27	*01	A234G	6	
TRAV30	*05	G127A	1	
TRAV36/DV7	*01	T186A	1	
TRAV39	*01	G47A	2	
TRAV40	*01	C77T	1	
TRAV6	*02	C147T	7	
TRAV8-2	*03	C177T	2	
TRBV12-4	*01	C227A	1	
TRBV12-5	*01	C28G	5	✔
TRBV19	*01	A24G	3	✔
TRBV30	*01	G259A	1	
TRBV5-1	*01	G42A	1	
TRBV5-4	*01	G258A	1	
TRBV5-6	*01	T245G	1	
TRBV6-6	*01	C222T	1	

Supplementary Table 4: Alleles inferred from the long read TCRseq data as described in the Methods and Supplementary Figure 6. The 'Inferred Allele' column indicates the nucleotide substitution required to generate the inferred allele from the nearest known equivalent in IMGT ('Closest Allele'). 'Number of donors' records in how many of the healthy volunteers' TCR repertoires the indicated novel allele was observed. The final column records whether a given allele was identified in the analysis of Omer & Peres *et al.* (Genome Medicine, 2022, 14:2, DOI: 10.1186/s13073-021-01008-4), which only considered TRB chain data.

Supplementary Figures

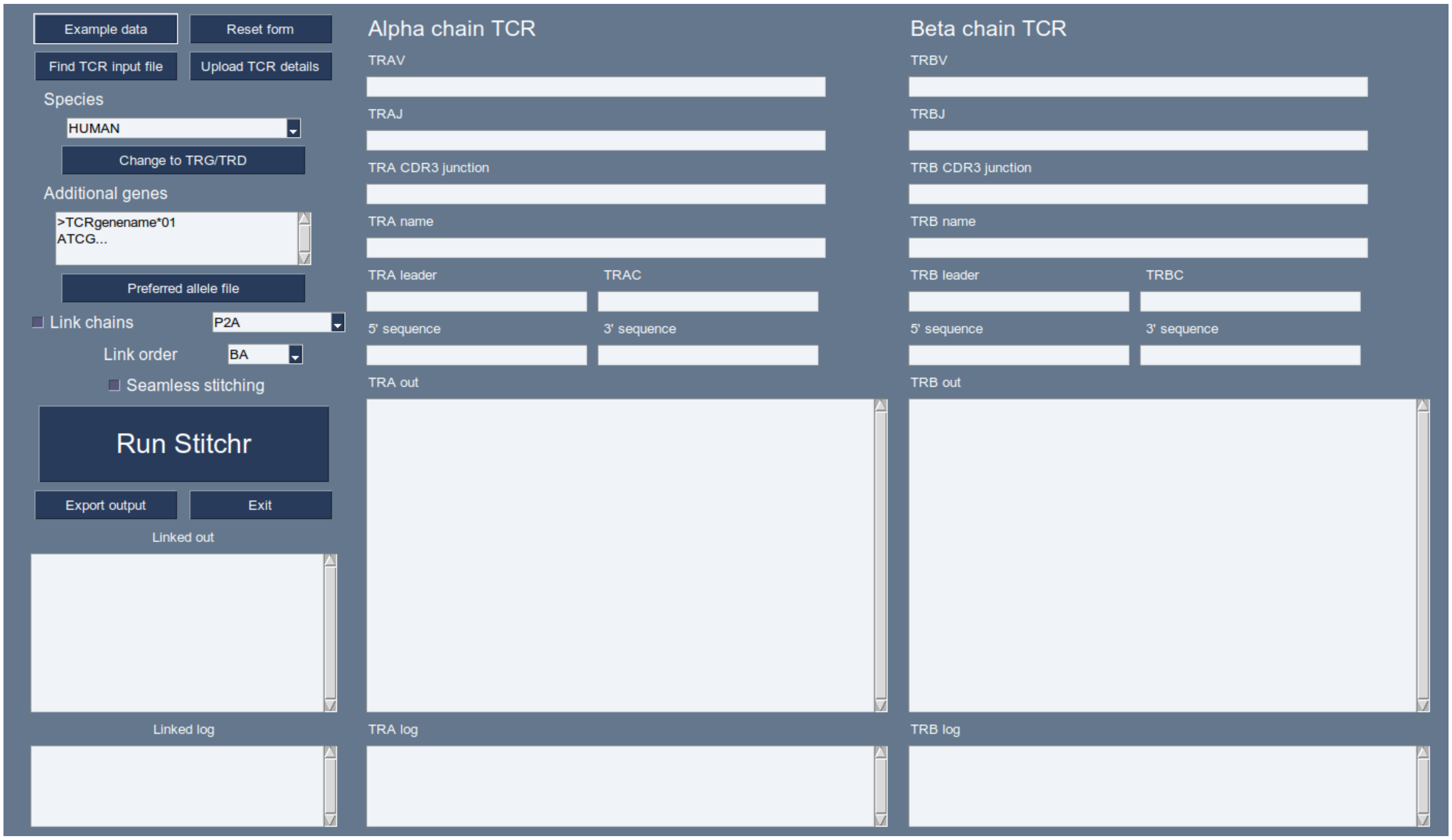

Supplementary Figure 1. Screenshot of the alternative graphical user interface to run Stitchr at low-throughput.

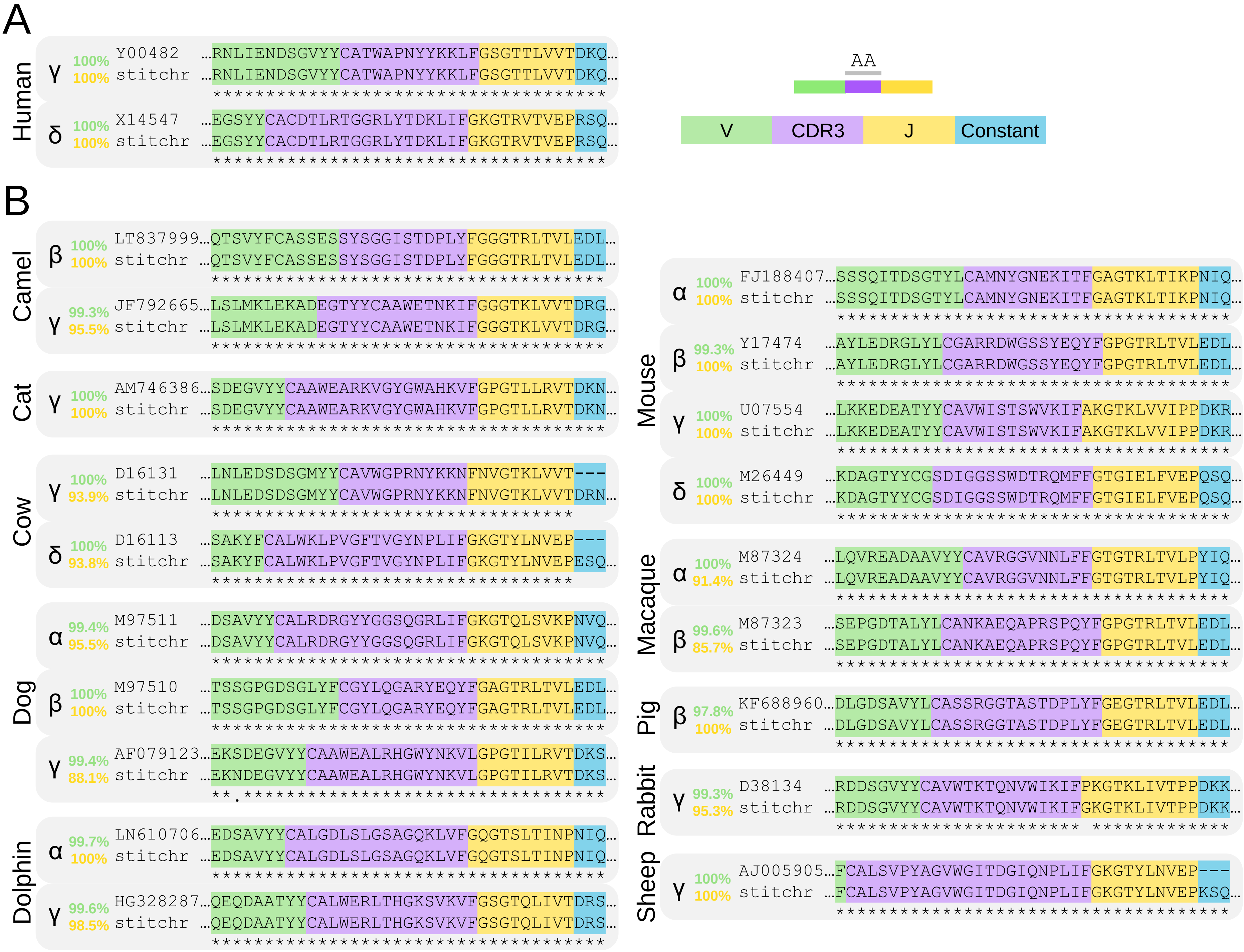

Supplementary Figure 2. Validating Stitchr on all TCR loci across species. Stitchr was used to reproduce human gamma/delta chain TCRs (**A**), as well as TCRs of all loci (alpha/α, beta/β, gamma/γ, and delta/δ) for as many species as possible given the data publicly available (**B**). Rearranged TCRs of the appropriate locus were identified by searching GenBank via the NCBI Nucleotide webportal, with a search string in the format [*genus species*] ["TCR" or "T cell receptor"] [*TRX* or *chain* (e.g. "TRA" or "alpha")]. Additional criteria used were to look only for mRNA sequences between 200 and 2000 nucleotides in length. TCRs were searched for all species/loci combinations for which IMGT currently provides at least one entry for each sequence type required for stitching (leader, variable, junctional, and constant regions), as shown in Supplementary Table 3. All combinations of species plus locus that the NCBI search returned rearranged TCRs for are featured here, with one example TCR chosen for each. Rearranged TCR were identified by submitting the FASTA entry to IMGT/V-QUEST, and the V/J/CDR3 (amino acid) information produced were provided to Stitchr via the command line. The Stitched protein sequence produced was then aligned against the translated original mRNA sequence (labeled with their GenBank accession), and aligned using Clustal Omega. The hypervariable region is shown here, colored by residue origin (variable gene residues green; CDR3 residues purple; junctional residues yellow; constant region residues blue). Colored text percentages to the left of alignments (e.g. 100% 100%) indicate the degree to which the input V (green) and J (yellow) genes matched the annotated gene as determined by V-QUEST, with non-100% values potentially indicating alleles not featured in the reference IMGT/GENE-DB database. Hyphens in the NCBI sequences indicate partial mRNAs that do not contain constant regions. Species names are: *Camelus dromedarius* (dromedary camel); *Felis catus* (cat), *Bos taurus* (cow); *Canis familiaris* (dog), *Tursiops truncatus* (bottlenose dolphin); *Homo sapiens* (human); *Mus musculus* (house mouse); *Macaca mulatta* (Rhesus macaque); *Sus scrofa* (pig); *Oryctolagus cuniculus* (European rabbit); *Ovis aries* (sheep).

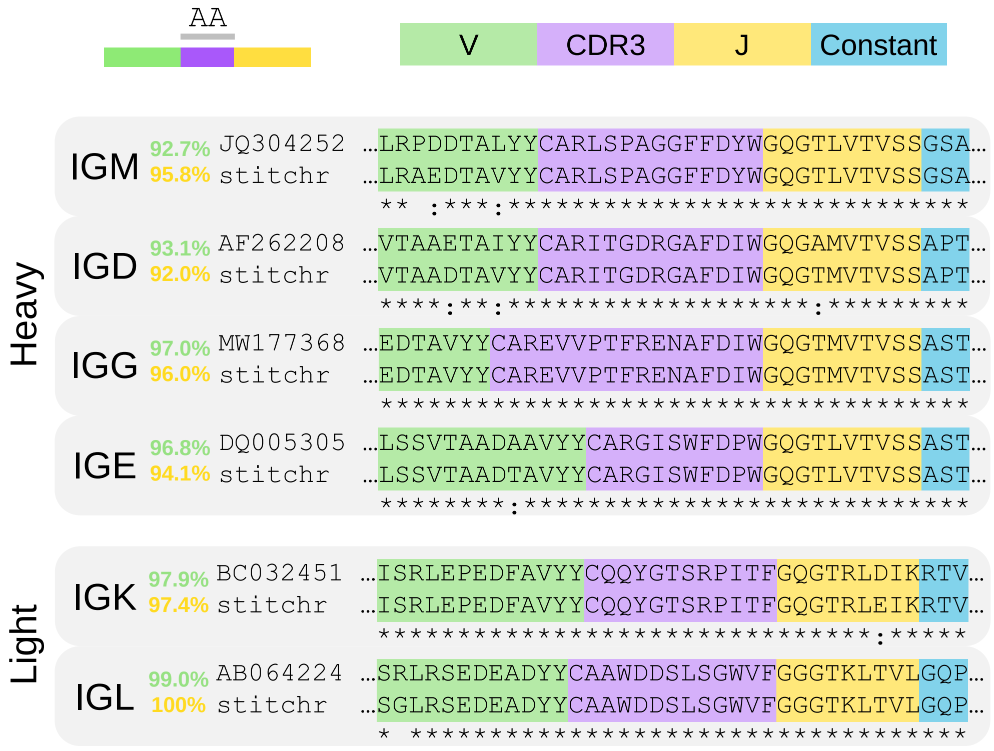

Supplementary Figure 3. Validating Stitchr on human immunoglobulin loci. Example rearranged IGs of the appropriate locus/class were identified by BLASTing the 5' end of a corresponding constant region against nt/nr and selecting mRNA between 200 and 2000 nucleotides in length. Rearranged IG were identified by submitting the FASTA entry to IMGT/V-QUEST, and the V/J/CDR3 (amino acid) calls produced were provided to Stitchr via the command line. The Stitched protein sequence produced was then aligned against the translated original mRNA sequence (labeled with their GenBank accession) using Clustal Omega. The hypervariable region is shown here, colored by residue origin (variable gene residues green; CDR3 residues purple; junctional residues yellow; constant region residues blue). Colored text percentages to the left of alignments (e.g. 100% 100%) indicate the degree to which the input V (green) and J (yellow) genes matched the V-QUEST called gene, with non-100% values potentially indicating alleles not featured in the reference IMGT/GENE-DB database or somatic hypermutation.

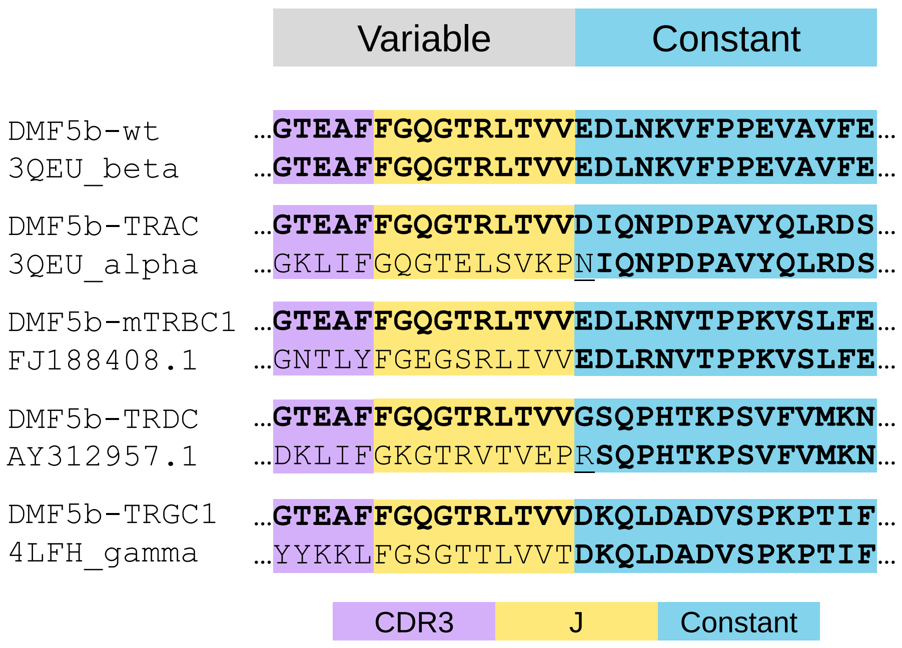

Supplementary Figure 4. Demonstration of Stitchr's utility in TCR engineering, through constant region domain swapping. The anti-MART1 TCR DMF5 beta chain (DMF5b) was stitched to a variety of constant regions (via the 'extra genes' function), and then aligned against various rearranged TCRs that incorporate the same constant region. The variable:constant domain interface is shown. From top to bottom, the stitched TCR/reference pairs are: DMF5b with its own TRBC1 constant region (DMF5b-wt) against its own PDB FASTA used to identify the V/J/CDR3 information (3QEU_beta); DMF5 with the alpha chain constant region (DMF5b-TRAC) aligned to the alpha chain of the DMF5 TCR (3QEU_alpha); DMF5b with a murine beta constant region (DMF5b-mTRBC1) aligned to a rearranged mouse beta chain cDNA (GenBank accession FJ188408.1); DMF5b with the delta chain constant region (DMF5b-TRDC) and a rearranged human delta chain (GenBank accession AY312957.1); DMF5 with a gamma constant region (DMF5-TRGC1) aligned to a rearranged gamma chain TCR (PDB accession 4LFH). Sequences matching the expected wild-type DMF5b sequences are in bold. Underlined residues indicate an expected amino acid mismatch: the first nucleotide of the first codon of the constant region is donated by the last nucleotide of the J gene post-splicing, thus the amino acid encoded will vary depending on the J gene used.

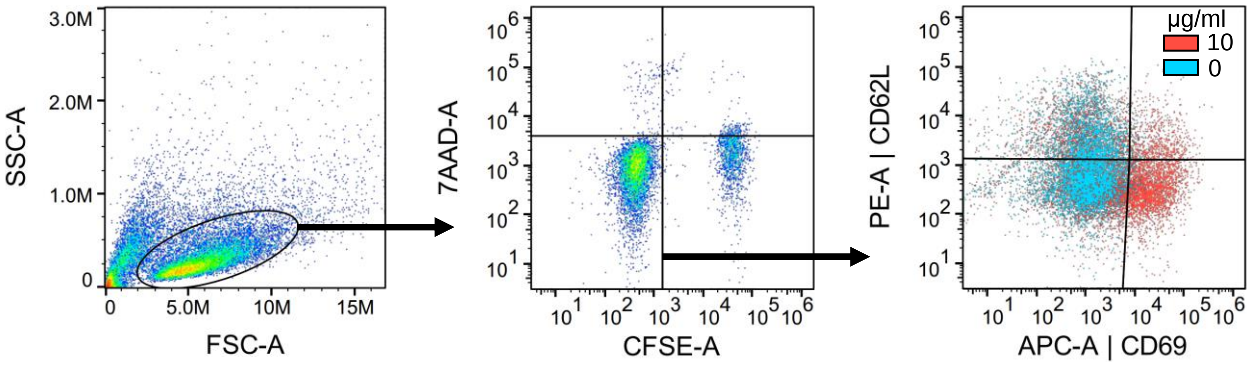

Supplementary Figure 5. Gating strategy of TCR-transduced Jurkat activation assay. Jurkat cells lentivirally transduced with a TCR of known reactivity were incubated overnight at a 2:1 ratio with CFSE-labelled peptide-pulsed target cancer lines either with matched or mismatched HLA-I alleles. Left to right: cells are gated away from debris, then singlets selected via FSC-A vs FSC-H (not shown), then live Jurkats gated on by taking CFSE- and 7AAD-negative cells. The frequency of activated Jurkats was determined by the percentage of CD69+ CD62L-negative cells.

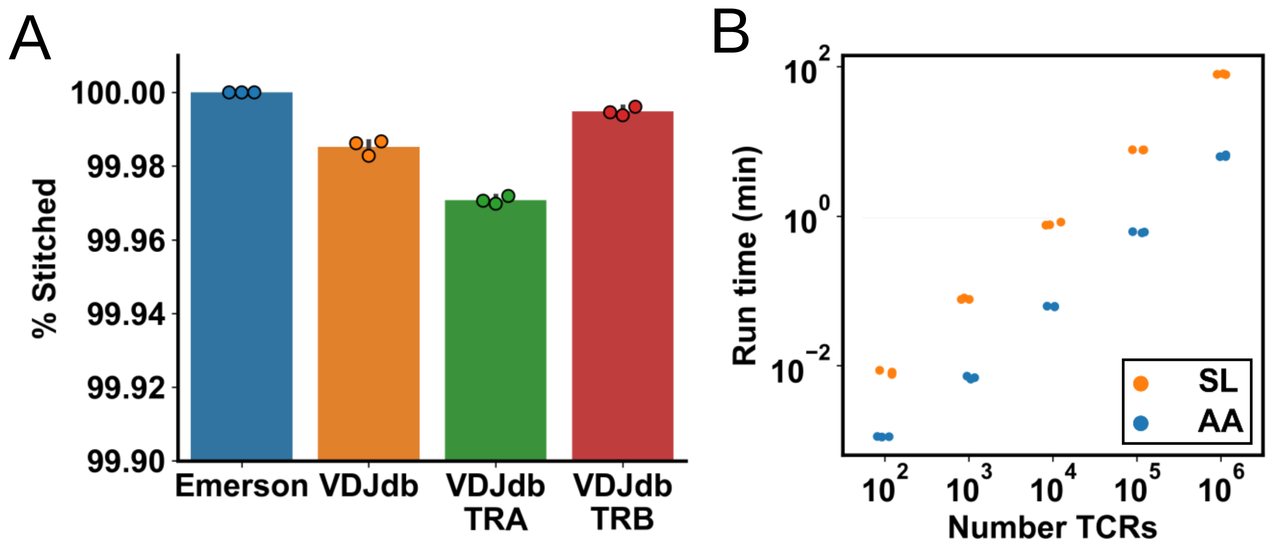

Supplementary Figure 6. Thimble performance running on published TCR repertoire datasets. **A,** Percentage of TCRs for which a stitched sequence was successfully produced (note cut Y axis lower bound). The Emerson data is a combination of five randomly chosen ImmunoSEQ beta chain runs, while VDJdb is the entirety of the human section of that database, further then split out into alpha (TRA) or beta (TRB) rearrangements only. Repertoires were run in triplicate, having up/down-sampled to different numbers. The results shown represent the percentage of stitched results from 1e6 TCRs. **B,** Comparison of run times for the Emerson data, up/down-sampled to different numbers of TCRs (X axis) when providing the CDR3 junction either as the junction-only amino acid sequence (AA) or the entire ~90 nucleotide sequence of the read (as listed in the 'rearrangement' column of the data) with the seamless stitching mode (SL) enabled.

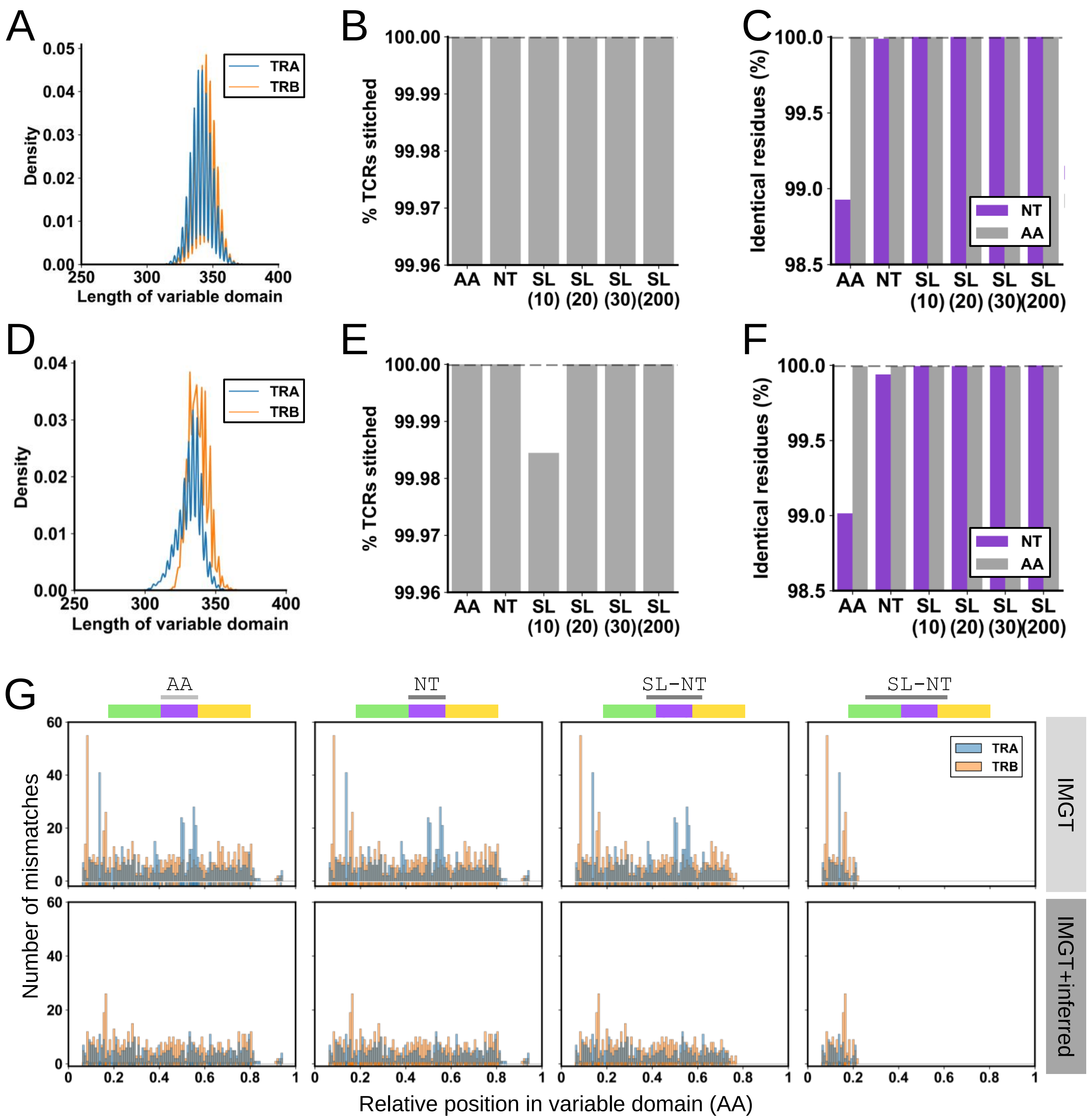

Supplementary Figure 7. Performance of Stitchr applied to high-throughput datasets. **A,** Density plot of variable domains (start of V-REGION to end of J-REGION) of TCRs produced by immuneSIM. **B,** Percentage of immuneSIM TCRs that successfully produced a stitched sequence. **C,** Percentage of residues (nucleotide in purple, amino acid in gray) that are identical between the input immuneSIM and output Stitchr/Thimble sequences. **D-F,** As in **A-C**, but for empirically sequenced TCRseq datasets from prior publications. **G,** Distribution of amino acid mismatches between translated sequences of TCRseq data when processed using just the typical IMGT reference database (top) versus IMGT supplemented with potential novel alleles inferred from the donors in the cohort (bottom), comparing different junction inputs (left to right: AA; NT; seamless 20-20; seamless 200-30).

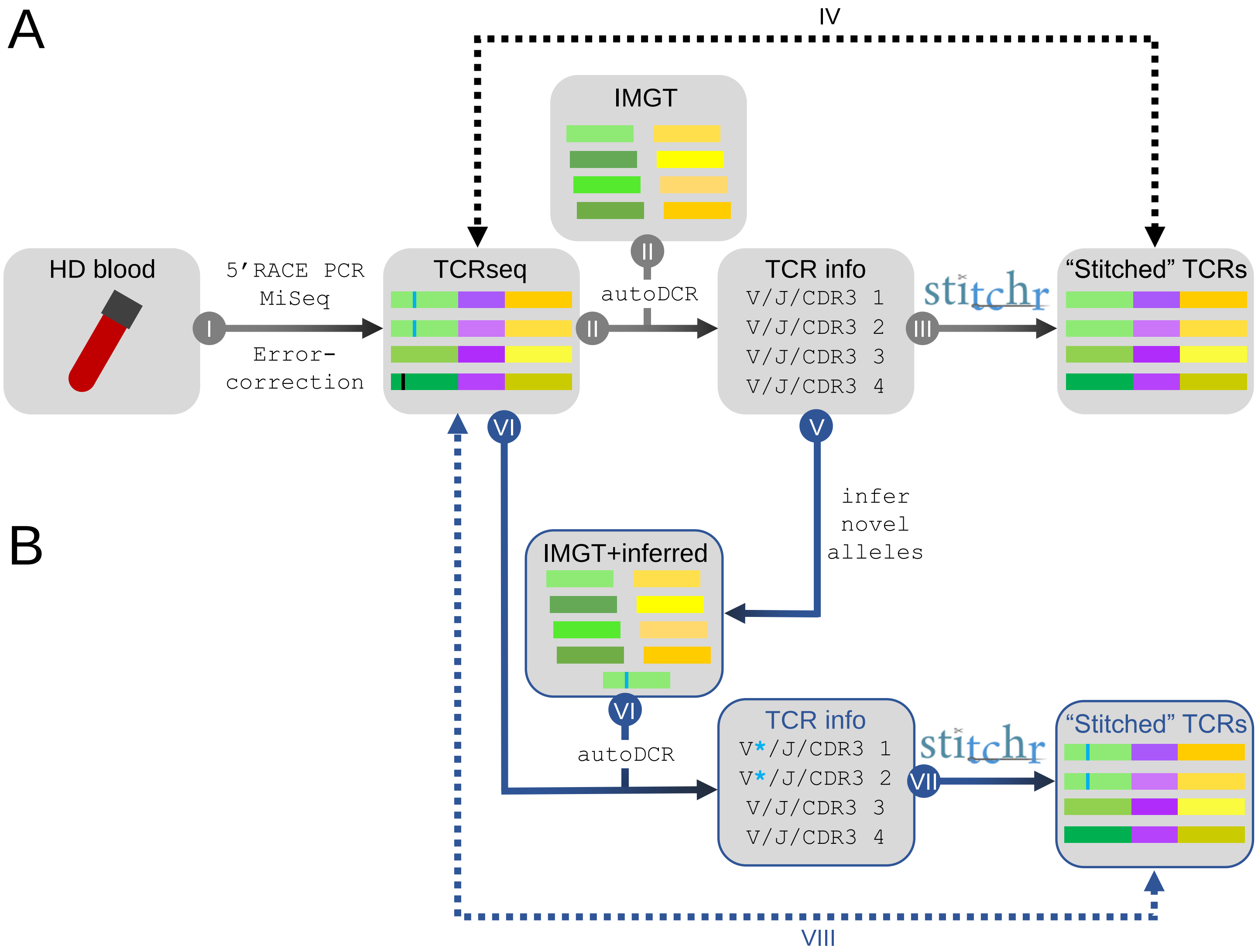

Supplementary Figure 8. Overview of Stitchr validation on real-world high-throughput TCRseq data. **A,** TCRs were sequenced from healthy donor peripheral blood RNA using a UMI-based 5’RACE protocol, sequenced on an Illumina MiSeq, and overlapping paired-ends were merged and error-corrected (I, see methods). Initially, TCRs in long error-corrected reads were annotated using autoDCR supplied with the IMGT reference database of V/J genes (II). TCR annotations produced were input to Stitchr (III, via Thimble), and sequences produced were compared to the sequences of the original reads used (IV). **B,** Alternatively, TCR information called by autoDCR was used to infer potential novel TCR V gene alleles, which were then added to the IMGT reference (V), which were then again used to annotate the original corrected reads with autoDCR (VI). Stitched TCRs produced with the IMGT+inferred reference (VII) were then compared to the original TCR sequences (VIII). Note that this process now accurately introduces SNPs contained in the novel TCR alleles (*), but will fail to replicate other mismatches between the read and the reference (e.g. sequencing errors) if they fall outside the range of a provided junction sequence.
